# Semi-automatic puncture robotic system based on real-time multi-modal image fusion: preclinical evaluation

**DOI:** 10.1007/s11548-025-03471-5

**Published:** 2025-08-14

**Authors:** Bo Zhang, Kui Chen, Yuhang Yao, Bo Wu, Qiang Li, Zheming Zhang, Peihua Fan, Wei Wang, Manxia Lin, Xiang Jing, Shigeki Sugano, Masakatsu G. Fujie, Ming Kuang

**Affiliations:** 1 WuXi AMIT Intelligent Medical Technology Col., Ltd., Wuxi, 214000 China; 2 Future Robotics Organization, Waseda University, Tokyo, 1620044 Japan; 3 The First Affiliated Hospital of Sun Yat-Sen University, Guangzhou, 510000 China; 4 Tianjin University Central Hospital, Tianjing, 300000 China; 5 Faculty of Science and Engineering, Waseda University, Tokyo, 1698555 Japan

**Keywords:** Puncture system, Liver puncture, Needle insertion, Surgical robot, Animal experiment

## Abstract

**Purpose:**

Traditional surgical robot system relying on computed tomography (CT) navigation suffers from two drawbacks during abdominal organ puncture surgeries. Firstly, the puncture target is displaced under the influence of respiration, thereby reducing the puncture accuracy. Secondly, the puncture process lacks real-time visualization, which may potentially give rise to medical accidents. This paper presents a semi-automatic surgical robot system based on the fusion guidance of real-time ultrasound images and CT, along with the monitoring of the patient’s respiratory state, to address these issues.

**Method:**

This system utilizes a six-axis force sensor in contact with the human body, and a respiratory model can be constructed through data got from force sensor to monitor the patient’s respiratory phase and recommend the optimal puncture phase. The issue of non-real-time puncture guidance is addressed through the real-time registration and fusion of ultrasound (US) images with preoperative CT images.

**Results:**

Phantom experiments and animal experiments were carried out based on this design. The test results indicate that in these two experiments, the average fusion error between US and CT of the main tissues in the liver is within 3 mm. For puncture accuracy, in the phantom experiment, the average puncture error was 1.0 mm, with a minimum of 0 mm and a maximum of 2.1 mm. In the animal experiment, the average puncture error was 2.5 mm, ranging from a minimum of 1.6 mm to a maximum of 3.0 mm.

**Conclusion:**

The results of two experiments show both the image fusion accuracy and puncture accuracy of this system are within 3mm, which can meet the requirement of 5 mm puncture accuracy in clinical practice. Approximately 70% of the operation are automatically accomplished by robot system, greatly reducing the reliance on the doctor’s experience.

## Introduction

The application and development of robotic technology in the medical field have introduced minimally invasive and high-precision treatment options [1–3], reducing the requirements for doctors’ skills and the dependence on experience in surgeries, and also improving the quality of life of patients. Among various types of cancers, the incidence rate of liver cancer ranks prominently on a global scale and is a leading cause of mortality threatening patients’ lives in China. Therefore, minimally invasive high-precision examination and treatment for liver tumors are of great significance for improving the overall health level and quality of life of people.

Liver will be pushed by the lungs and diaphragm during breath, and the displacement in the head-to-foot direction can reach about 4 cm [4]. The liver itself will also be squeezed and deformed. To accurately locate the tumor, one of the traditional solutions refers to the strategy of skeletal robots, adopting the technical path of optical positioning sensors plus CT. It locates the tumor position of the patient via markers affixed to the patient’s abdomen to achieve registration with the CT image. Simultaneously, the patient’s respiration is tracked, and the puncture phase is recommended through a respiration monitoring module [5, 6]. Another solution, also based on CT guidance, depends on the relative positional relationship between the robot and the CT equipment for positioning, and the robot must be positioned in the calibrated location beside the CT [7]. It monitors the respiratory phase by means of a breathing belt fastened to the patient’s abdomen and executes the puncture during a specific respiratory phase. The above surgical robot systems have significant defects. Firstly, during the puncture surgery under the guidance of CT, the real-time state of the puncture needle and the relative positional relationship between the puncture needle and various liver tissues are invisible to the doctor, which may lead to surgical accidents. Secondly, the optical positioning sensors used for respiratory tracking may be blocked, and the markers may not be visible, which will lead to the failure of the tracking function. Thirdly, doctors are restricted to performing surgeries within the CT room, which curtails the application and dissemination of such robots. The use of mechanical sensors can solve the problem of the optical positioning sensors being blocked. Some scholars have already carried out research and adopted the scheme of using wearable strain sensors to conduct force monitoring and build respiratory models [8, 9]. However, the problem with this solution is strain sensors need to be attached to the patient’s abdomen all the time and may interfere with the position of the puncture surgery.

We developed a puncture system [10]to solve part of the above-mentioned problems, and the puncture accuracy of it has been verified. However, this system does not have respiratory monitoring function. In this paper, a respiratory monitoring module was introduced to solve the remaining other problems.

## System design

**Fig. 1:**
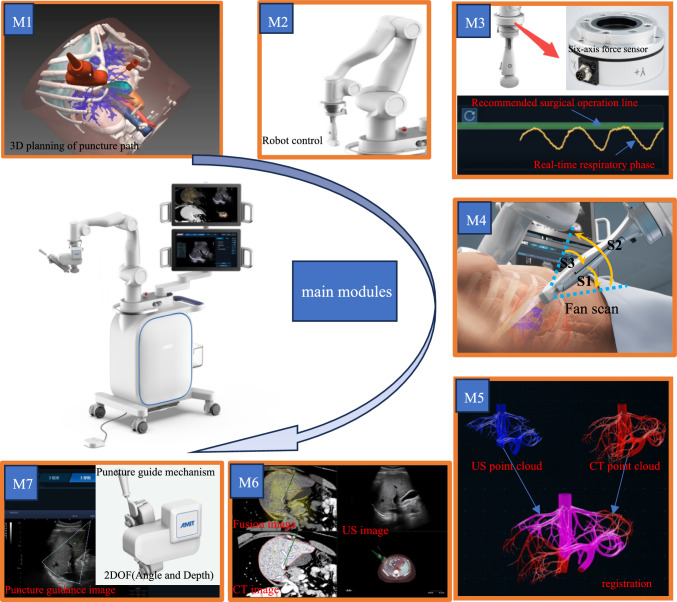
Robot design and main modules

To achieve the expected functions, this robot system has constructed the following modules: preoperative planning module (M1), motion control module (M2), respiration monitoring module (M3), US image reconstruction module (M4), image registration and fusion modules (M5 and M6), and puncture guidance module (M7), as shown in Fig. 1. With the collaborative operation of these modules, the robot system is able to possess the capabilities of precise tumor localization, real-time US image fusion, and adaptive puncture guidance. The primary functions and technical characteristics of each module are detailed as follows:

### Preoperative planning module (M1)

This module is activated before the start of the operation to facilitate doctors to pre-formulate the surgical plan. Doctors can use preoperative planning software to analyze the imported CT images, use the internal tools to segment key tissues such as the liver, internal blood vessels, tumors, and gallbladder. Then, doctor sets US probe placement position and the appropriate puncture needle path based on segmented results, then exports the results of preoperative planning.

### Motion control module (M2)

The core hardware of this module is a 7 degrees of freedom (DOF) robotic arm, and its end is rigidly connected to the US probe, and it has two working modes. The first is the free dragging mode. The doctor can freely manipulate the robotic arm by holding the US probe and place the US probe in any working space. The second is the active motion mode. The robotic arm will automatically execute actions such as fan scan or reach the specified position. The system can use the positional data of the robotic arm and the information of US images to replace the external optical sensors of traditional surgical robots for tumor localization.

### Respiration monitoring module (M3)

The key hardware of this module is a high-precision six-axis force sensor, which is installed between the robotic arm and the US probe. Its function is to collect the force signal and transmit it to the robot system. The system constructs a respiratory model based on this force signal and gives a recommended operation phase for precise puncture. During CT scanning, to reduce the impact of respiration, the operator will ask the patient to hold their breath for a moment after inhaling. Theoretically, when scanning and puncture the liver in this respiratory state, the similarity between the constructed US image and the CT image is the highest, the deformation and difference of each organ are the smallest, and the highest puncture accuracy can be obtained at this time.

Its main principle is to establish a mathematical correlation between the periodically changing force applied to the sensor and the respiratory phase. In this system, by using the six-axis force sensor connected to the probe, the force applied to the US probe is obtained to construct a respiratory phase model, and a suitable surgical operation line is recommended according to the respiratory state. When the patient’s respiratory state is close to the green surgical operation line, it is the theoretically optimal puncture phase. In the process of constructing the respiratory model, the respiratory states at the end of inspiration and the end of expiration have the greatest impact on the model. By collecting these data and conducting statistical analysis, the recommended respiratory phase for surgery is provided. Therefore, even if the respiratory patterns of different individuals vary, this model can be fully applicable. 1,4 The six-axis force sensor also plays a crucial role in ensuring system safety. Once the contact force of the probe surpasses a predefined threshold, the system will be promptly halted, thus effectively preventing potential accidents.

### Three-dimensional image reconstruction module (M4)

System will control the US probe to perform a fan scan, get the US information of the liver tissue in the area passed by the probe. The reconstruction algorithm uses the obtained US images and the position data of the robotic arm to construct a three-dimensional(3D) US model. Then convert the real-time US blood vessels into a point cloud. The 3D US model can be used to establish the relationship between the real human tissue (the image obtained by US) and the preoperative CT.

### Image registration (M5) and fusion modules (M6)

This module registers the acquired 3D US images and CT images based on the point cloud of blood vessels. The intrahepatic blood vessels can be distinctly visualized under CT and US modalities, while the visualization of tumor under US is sometimes not good. However, the relative position of the intrahepatic blood vessels and the tumor is fixed. Hence, the tumor position can be calculated more accurately based on the registered blood vessels. This system adopts feature-based point cloud registration method [10] based on characteristic of intrahepatic blood vessels. During the puncture operation, the US probe will continuously be in contact with the human skin to collect US image information inside the liver. The fused CT and US images are displayed in real-time, enabling the doctor to perform precise puncture operations in the two-dimensional US image, and observe in the 3D CT image.

### Puncture guidance module (M7)

This module mainly consists of a two DOF puncture guide mechanism and its control system. Based on the results of preoperative planning(M1) and image registration(M5), the system can accurately locate the tumor and automatically deliver the US probe to the planned position through the robotic arm. After the doctor installs the puncture guide mechanism, it will automatically adjust the puncture angle and depth to prepare for puncture. This guidance module can provide a stable puncture support for the doctor ensure that the puncture needle is always within the US plane during the puncture process and move along the preset guideline. Meanwhile, to fully incorporate the doctor’s clinical experience, the doctor is permitted to adjust the surgical path during the operation. By simply selecting the position to be punctured on the US image, the puncture guidance module will automatically modify the puncture guidance angle and depth according to the selected position, thereby fulfilling the operation in accordance with the doctor's requirements.

## Surgical robot system workflow

**Fig. 2:**
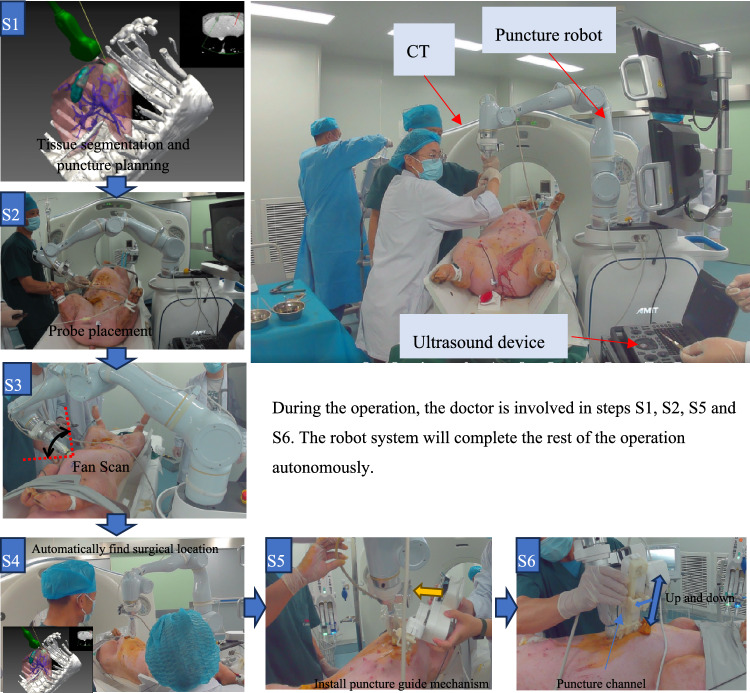
Operational procedures

The operation process of this robot system is simple and conforms to the doctor’s operation habits. During the implementation of the puncture operation (excluding preoperative planning), 70% of the entire operation process is automatically executed by the robot, and the doctor only needs to participate in part of the operation. The entire process is shown in Fig. 2, and the specific steps are as follows:

### Preoperative planning (S1)

This step can be accomplished in advance. The doctor selects the appropriate placement position for the probe and the entry path of the puncture needle, while avoiding key organs such as blood vessels and gallbladder. Eventually, a surgical plan is generated and saved into a USB flash drive. Before the start of operation, the doctor import the prepared surgical plan into this surgical robot system from the USB flash drive. This system adopts a dual-monitor layout, with the upper and lower monitors arranged. The upper monitor mainly displays the CT and the US fusion image, and the lower monitor exhibits the surgical operation process.

### Placement of the US probe (S2)

The doctor put the US probe near the area rich in liver blood vessels. This step is to pave the way for the next operation (fan scan). It should be noted that this placement position of the US probe does not have to be the same as that in the preoperative plan.

### Registration and fusion of US and CT data (S3)

When the respiratory phase is close to the recommended surgical operation line, the doctor communicates with the patient to make them hold their breath temporarily. The robotic arm drives the probe to perform a fan scan. The fan scan angle is ± 15 degrees, and the duration is 10 s. During this period, the US image is transmitted to the control system in real-time, and robot system builds a 3D US blood vessel point cloud. Then the robot system automatically registers the constructed US blood vessel point cloud with the CT blood vessel point cloud, thus realizing the real-time fusion of US and CT to guide the puncture operation. The registration time will vary slightly depending on the number of ultrasonic point clouds obtained from 3D reconstruction, ranging from 25 to 35 s, with an average time of 30 s. During the registration process, patients do not need to hold their breath and can breathe freely. After registration, the CT images and US real-time images will be fused immediately.

### Automatic movement of the robotic arm (S4)

Based on the placement position of the US probe in the preoperative plan and the registration results of the US and CT data, the doctor only needs to step on the foot pedal to send an autonomous movement control signal. The robot system will then control the robotic arm to carry the US probe and automatically reach the preset position of the US probe.

### Installation of the puncture guide mechanism (S5)

The doctor manually installs the puncture guide mechanism. After a specific initialization process, the puncture guide automatically adjusts the puncture depth and angle according to the puncture path in the preoperative plan and the registration results.

### Monitoring of respiration and implementation of puncture (S6)

The doctor refers to the respiratory phase monitor. When the real-time respiratory curve is close to the operation line, the patient holds their breath. The doctor confirms that the puncture path is appropriate on the US image based on the fused image, and then inserts the appropriate needle completely along the puncture guide mechanism.

## Experiment

The purpose of this experiment is to test image fusion error and the puncture error and of this system. According to the relevant requirements for liver ablation treatment [11], a safety margin of 5 mm is needed. Hence, the maximum image fusion error and puncture error should not exceed 5 mm. This paper carried out phantom and animal experiments in accordance with the workflow introduced above.

### Experimental site

CT Room: In fact, this system does not depend on the CT room. However, to facilitate the CT scan after the experiment to evaluate the puncture accuracy, these experiments were carried out in a CT room. The ultrasonic equipment (SonoScape X5, Shenzhen Sonoscape, China) and 14-gauge trocar (XD 2.1*200, Zibo Mingyuan Industry, China) were used in these experiments.

### Test objects

CIRS 057A phantom (including liver, blood vessels, and bones) and three pigs, as shown in Table 1.

**Table 1:** Experiment settings

Method	Subjects	Implanted targets	Puncture times for each target	Additional information
Phantom experiment	Abdominal phantom	4	3	CIRS 057A
Animal experiment	Pig 1	4	1	50 KG,12 months old
	Pig 2	4	1	52 KG,12 months old
	Pig 3	4	1	51 KG,12 months old

## Experimental procedure

The principal steps and procedures of the two experiments are detailed as follows.

### Experimental preparation

In the case of the phantom experiment, an US examination was conduct to ensure that the phantom is not damaged. The experimental pigs were fasted but not deprived of water 12 h before the operation. The animals were under general anesthesia during the operation, and the respiration was maintained by a ventilator. During this period, the respiratory rate, heart rate, and blood oxygen saturation were continuously monitored.

### Implantation of target points

**Fig. 3:**
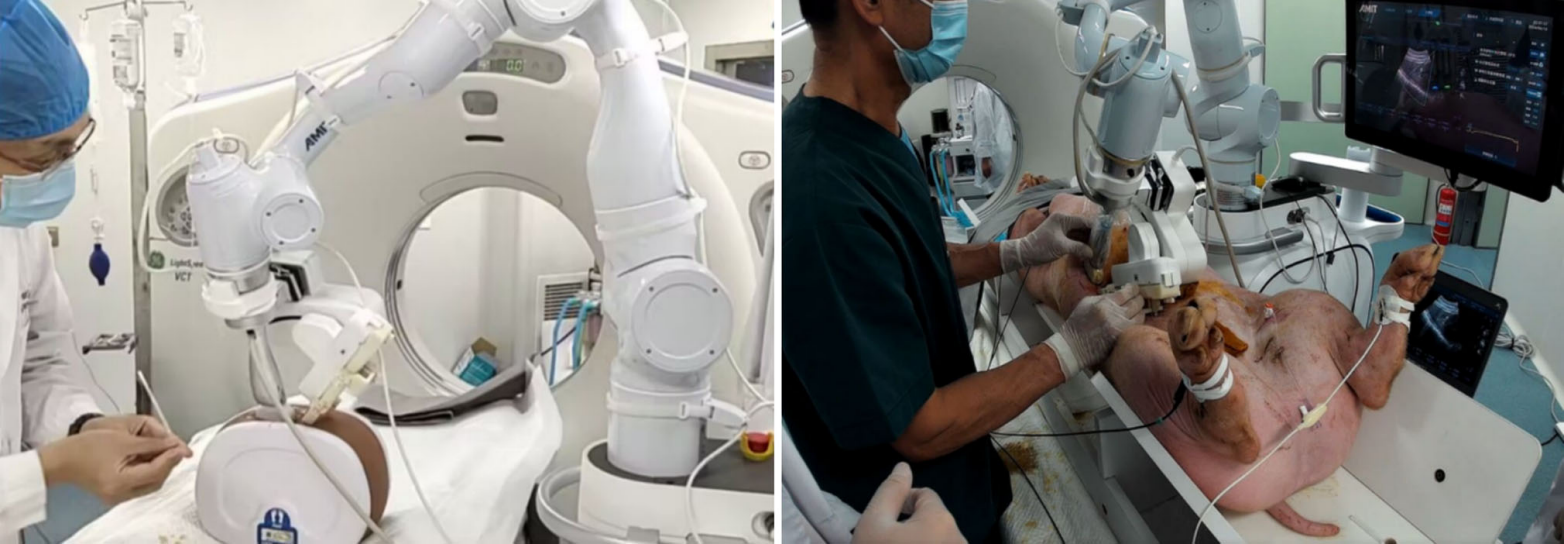
Phantom experiment and animal experiment

Under the guidance of real-time US, pre-made 1 × 3 mm titanium particles w implanted into the liver using a 14G trocar needle to form the puncture target. For the phantom, a total of four titanium particles were implanted into the liver. Each pig was implanted with four titanium particles. The research findings of some researchers indicate that tumors close to blood vessels and those in deeper positions have relatively higher puncture difficulties [12, 13]. Therefore, among the four titanium particles designed in this experiment, at least one should be within 5 mm from the blood vessel, and another one should be implanted into the deeper part of the liver (Fig. 3).

### CT scanning

The CT scanning parameters were set as a layer thickness of 0.625 mm and a layer spacing of 0.625 mm. For the phantom, a normal CT scan was sufficient. For pigs, to make the blood vessels clearly visible, an enhanced CT scan was required. At the end of inhalation, the operator paused the ventilator during scanning and immediately resumed the ventilator operation after the scan was completed to maintain the stable breathing state of the pig.

### Surgical planning

**Fig. 4:**
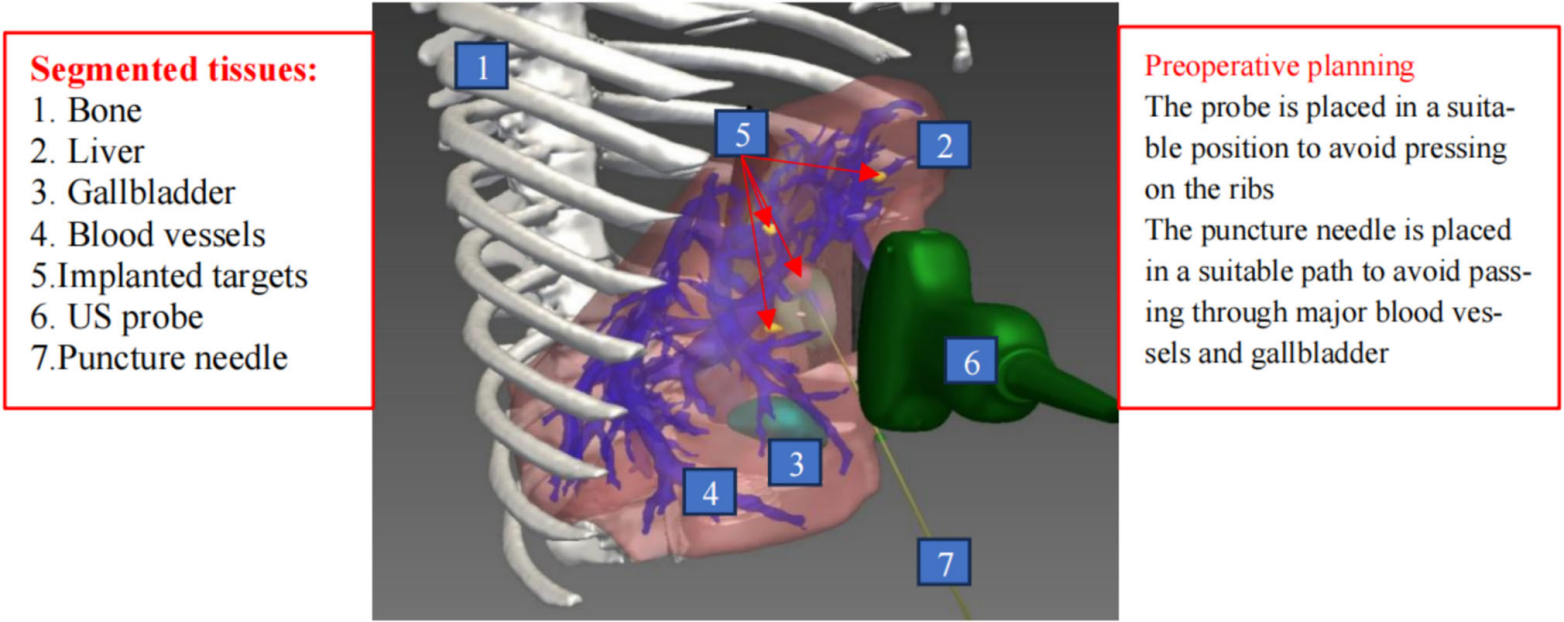
Pig liver tissue segmentation results and preoperative planning

The doctor carried out preoperative planning according to the procedures. The titanium particle targets are shown as yellow particles in Fig. 4. For the phantom, three puncture paths were planned for each puncture target; while for pigs, one puncture path was planned for each puncture target.

### Surgical navigation

The doctor carried out surgical navigation according to the operating procedures. Given that the pig's skin was thick and hard, and the puncture needle was not easy to pierce, after installing the puncture guide mechanism, a skin-breaking knife was used to break the skin at the needle insertion site.

### Respiratory control and puncture

When the real-time respiratory curve was close to the operation line, the doctor instructed the assistant to pause the operation of the pig's ventilator. The doctor confirmed that the puncture trajectory was safe and correct on the fused US image, and then quickly inserted the sterilized 14G trocar needle according to the angle and depth limited by the puncture guide mechanism. It only takes few seconds to finish needle insertion. Then, the torcar needle was fixed to reduce the positional deviation caused by respiratory. During the operation, if the assistant fails to stop the ventilator phase at the recommended phase for the first time, the ventilator can be started and stopped again until the recommended position is reached. Meanwhile, in clinical application, if the phase at which the patient holds his/her breath is not at the recommended phase, the doctor can communicate with the patient to inhale and hold the breath again, so that the respiratory phase stays at the recommended phase to meet the requirements of the puncture.

### Puncture error evaluation

The experimental animals and phantom with needles were scanned by CT again, and the layer thickness and layer spacing were still set as 0.625 mm. The CT data was analyzed using 3D Slicer (an open-source medical image viewer widely used by doctors, scholars, and R & D personnel), and its measurement function was used to obtain the distance (puncture error) between the needle tip and the puncture target in the CT.

In the phantom experiment, there was no need to confirm the respiratory state, and the other operation procedures were the same as those in the animal experiment.

## Results

### Evaluation of image fusion accuracy

**Fig. 5:**
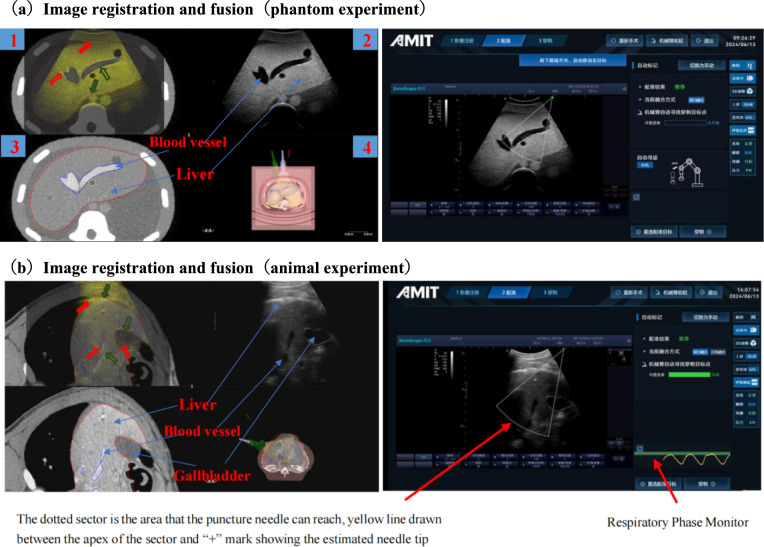
Image registration and fusion results

Since the phantom did not have respiratory movement, the respiratory monitoring module had no practical application in the phantom experiment. Figure 5 (a) illustrates the results of image registration and fusion in phantom experiment. The left figure shows the image displayed on the upper screen, where Region 1 is the result of CT and US fusion image, Region 2 is the real-time US image obtained by the probe, Region 3 is the CT image corresponding to the US image based on the registration result (the red line represents the preoperative segmented liver contour line, and the blue line represents the blood vessel contour line), and Region 4 is the 3D global view. From the fusion result of CT and US, the coincidence degree of the liver and vascular contours of the two was relatively high.

Figure 5 (b) shows the fusion result of CT and US images in animal experiment based on respiratory monitoring module. In the respiratory phase monitor box, the yellow curve is the real-time respiratory curve obtained by using a six-axis force sensor, and the green line is the recommended operation respiratory phase line by the system. Theoretically, at this phase, the US image should be able to well coincide with the intercepted CT image, and each tissue should be accurately aligned. Through the fusion image on the upper screen, it can be found that the coincidence of the edge contours of the liver, main blood vessels, and gallbladder corresponding to the CT and US images was good.

In Region 1 of the upper screen fusion image in Fig. 5, the fusion differences of the liver, gallbladder, and main vascular contours were measured. Among them, the part indicated by the red arrow was the area with poor fusion results, and the part indicated by the green arrow was the area with good fusion results. By utilizing the US measurement function, 6 sets of fusion deviations of each tissue contours in these areas were tested. The results indicated that the overall error of image fusion was within 3 mm, and the test results are presented in Table 2 in detail. Due to the large size of the liver and the existence of deformation, the overall fusion error is large. However, blood vessels are fixed inside the liver and have less deformation, so the fusion results are the best. Moreover, the characteristics of blood vessel branches are obvious. The registration based on the blood vessels inside the liver is conducive to improving the positioning accuracy of liver tumors.

**Table 2:** US and CT image fusion results

	Organ	Minimum deviation (mm)	Maximum deviation (mm)	Average deviation (mm)
Phantom	Liver	0.1	1.0	0.4
	Blood vessel	0.1	1.2	0.6
Pig	Liver	0.3	4.5	3.0
	Gallbladder	0.2	4.3	2.8
	Blood vessel	0.1	1.8	1.5

### Evaluation of puncture accuracy

**Fig. 6:**
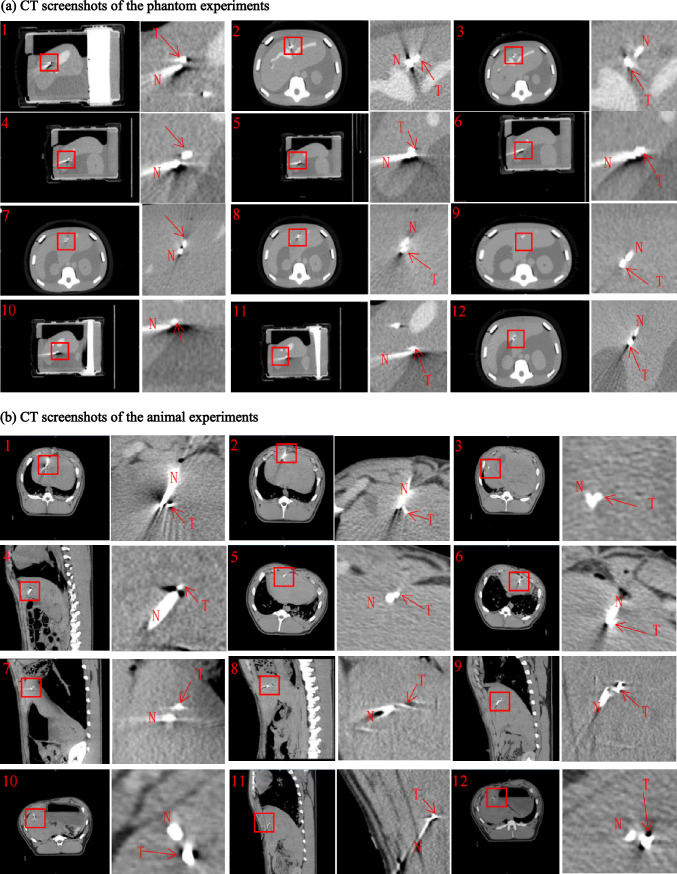
CT screenshot of the relationship between the puncture needle tip and the target point

The data in Table 3 show the phantom and animal experiment puncture error results obtained according to the experimental puncture error evaluation method. The CT screenshot of the phantom puncture result is shown in Fig. 6a, and the animal experiment puncture result is shown in Fig. 6b. These figure shows a section in the CT image where the needle tip and the target point can be simultaneously observed (one of the cross-sectional, sagittal, or coronal planes), as well as a partial enlarged view of the needle tip and the target point. In these pictures, N represents the puncture needle and T represents the puncture target.

**Table 3:** Puncture accuracy of phantom and animal experiments

No	Phantom (mm)	Animal (mm)
1	1.3	3.0
2	0.7	2.8
3	2.1	2.5
4	1.5	1.6
5	1.5	2.8
6	0	2.6
7	0.5	2.9
8	0	2.9
9	0.3	2.1
10	1.5	2.4
11	0.9	2.3
12	2.1	2.2
Average	1.0	2.5

The results indicates that average puncture error in the phantom experiment is 1.0 mm, the minimum puncture error is 0, and the maximum puncture error is 2.1 mm. In the animal experiment, the average puncture error is 2.5 mm, the minimum puncture error is 1.6 mm, and the maximum puncture error is 3.0 mm. A puncture error of 0 means that the tip of the puncture needle after 3D reconstruction was in contact with the punctured target. The test results indicate that, regardless of whether it is a phantom test or an animal experiment, the puncture error between needle tip and the planned puncture target does not exceed 5 mm, and such puncture accuracy can meet the clinical requirements. Regarding the puncture efficiency, regarding the time from fan-shaped scanning to the completion of puncture, the average completion time of the robot is 64.6 s (maximum 67 s, minimum 62 s) [10] in our previous research. For comparison, other researchers have shown that the needle insertion time for manual liver puncture is 6 to 15 min [14]. Obviously, the puncture efficiency of the robot is higher.

## Discussion

In the phantom and animal experiments, the designed robot system worked well, and no adverse events occurred in operation process during the experiment. Both the phantom and the animal experiment were successfully conducted, and the puncture accuracy fully met the clinical requirements, effectively solving the problems caused by the movement of the tumor position and the non-real-time puncture guidance in the clinic. The robot system has good mobility. It only requires one person to push the equipment to transfer between various departments in the hospital. Moreover, the system does not rely on the CT room during operation; it can be used in a general operating room, requiring only the import of the patient's preoperative CT scan data into the system. Overall, this surgical robot system has the following characteristics:

- *Reduced Dependence on Doctor’s Experience*: The system has a high degree of automation, and more than 70% of the surgical process can be automatically completed by itself. The system can automatically control the robotic arm to drive the probe for fan scan, complete image registration, take the probe to the preoperatively planned puncture target position, and adjust the puncture angle and depth of the puncture guide. Doctor only needs to place the probe in a position rich in blood vessels at the beginning of the operation and insert the puncture needle along the puncture guide mechanism after confirming that the puncture navigation is correct.
- *Improved Surgical Safety*: During the surgical procedure, if the registration error exceeds the expected value, the system will provide doctor with two operational options. The first is to restart the surgery; the second is to disregard the registration and fusion results. Based on the current ultrasound images and puncture guidance lines, the doctor can select the intended puncture site on the system interface according to their experience, and the puncture angle and depth will be automatically adjusted. The robotic arm can hold the US probe and provide a stable real-time US image. The structural design of the puncture guide mechanism ensures that the puncture needle is always within the range of the US image and can predict the trajectory of the puncture guideline. The doctor can ensure the safety of the puncture by observing the relationship between the tissue and the guide line in the US image, greatly reducing the risk of misoperation.

### Increased surgical precision

The adjustment of the puncture angle and depth in this system is relatively flexible. It cannot only automatically recommend the puncture angle and depth but also give the doctor a certain degree of freedom of operation. After the doctor manually selects the puncture target, the system can automatically calculate the corresponding puncture angle and depth and automatically make corresponding adjustments. Therefore, with the assistance of this system, the doctor can always quickly lock the puncture target and accurately complete the puncture operation.

However, the system also has certain limitations. Since no external position sensor is used, if the patient moves during the operation, the robot system cannot actively detect this movement and cannot perform compensation. In this case, the patient can only be re-fixed, and the doctor needs to re-perform fan scan scanning, registration, and other surgical procedures at the new position. However, the surgical efficiency of this system is relatively high. By operating according to the system process, the operation can be completed in only a few minutes. In the future, we will introduce machine learning into this system to perform deformable registration between intraoperative ultrasound images and preoperative CT images, aiming to solve the problem that the liver, due to respiratory-induced deformation, cannot be precisely fused with the preoperative CT. We will also expand the application scenarios of the robot and extend it to tissues such as the prostate and kindly.

## Conclusion

Phantom and animal experiments were carried out in this paper. The experimental results confirmed that with the help of this system, doctors can achieve a puncture accuracy within 3 mm, which can meet the clinical requirement of 5 mm puncture accuracy. Next, we will conduct clinical trials to verify the availability of this robot system.
