# Correction: Semi-automatic puncture robotic system based on real-time multi-modal image fusion: preclinical evaluation

**DOI:** 10.1007/s11548-025-03520-z

**Published:** 2025-10-15

**Authors:** Bo Zhang, Kui Chen, Yuhang Yao, Bo Wu, Qiang Li, Zheming Zhang, Peihua Fan, Wei Wang, Manxia Lin, Xiang Jing, Shigeki Sugano, Masakatsu G. Fujie, Ming Kuang

**Affiliations:** 1 WuXi AMIT Intelligent Medical Technology Col., Ltd., Wuxi, 214000 China; 2 Future Robotics Organization, Waseda University, Tokyo, 1620044 Japan; 3 The First Affiliated Hospital of Sun Yat-Sen University, Guangzhou, 510000 China; 4 Tianjin University Central Hospital, Tianjing, 300000 China; 5 Faculty of Science and Engineering, Waseda University, Tokyo, 1698555 Japan

**Correction to: International Journal of Computer Assisted Radiology and Surgery** 10.1007/s11548-025-03471-5

In the original version of this article, there were three corresponding author (namely Kui Chen, Xiang Jing, and Ming Kuang), but in the article, only Kui Chen was mentioned as corresponding author and missed to update Xiang Jing, and Ming Kuang as co-corresponding authors.

The original article has been corrected.

